# Gargling with Ketamine Attenuates the Postoperative Sore Throat

**Published:** 2009-02

**Authors:** A Rudra, Suchanda Ray, S Chatterjee, A Ahmed, S Ghosh

**Affiliations:** 1 Consultant Anaesthesiologist, Kolkata; 2 Assistant Professor of Anaesthesiology, Medical College & Hospital, Kolkata; 3 DNB Student, Apollo Gleneagles Hospital, Kolkata

**Keywords:** Complications, Sore throat, Intubation, Ketamine gargle

## Abstract

**Summary:**

Postoperative sore throat (POST) is a common complication of anaesthesia with endotracheal tube that affects patient satisfaction after surgery. Therefore, this complication remains to be resolved in patients undergoing endotracheal intubation. The aim of the study was to compare the effectiveness of ketamine gargles with placebo in preventing POST after endotracheal intubation. Forty patients scheduled for elective surgery under general anaesthesia were randomized into: Group C, water 30 ml; Group K, ketamine 50 mg in water 29 ml. Patients were asked to gargle this mixture for 40 seconds, 5 minutes before induction of anaesthesia. POST was graded at 4, 8 and 24 hours after operation on a four-point scale (0-3). In the Control group POST occurred more frequently, when compared with patients belonging to Ketamine group, at 4, 8, and 24 hours and significantly more patients suffered severe POST in Control group at 8 and 24 hours compared with Ketamine group (*P*<0.05). We demonstrated that gargling with ketamine significantly attenuated POST, with no drug-related side effects were observed.

## Introduction

Recently, quality assurance of anaesthesia has become increasingly important for improving postoperative outcome. Postoperative sore throat (POST) is a minor complication that is unresolved in patients undergoing endotracheal intubation^1^–^7^. POST was recently ranked by American anaesthesiologists as the eighth most important problem of current clinical anaesthesiology^8^. POST following tracheal intubation is due to trauma to the airway mucosa. The reported incidence of POST varies from 21 to 65%^2^,^9^,^10^. Various pharmacological and non – pharmacological trials have been used for attenuating POST with variable success. The pharmacological methods include beclamethasone inhalation and gargling with azulene sulfonate^11^,^12^.

It has been shown that NMDA receptors are present not only in the central nervous system but also in the peripheral nerves^13^,^14^. It has been further reported that peripherally administered NMDA receptor antagonists are involved with antinociception^15^ and anti-inflammatory cascade^16^.

In this study, we investigated whether preoperative gargling with ketamine, a NMDA receptor antagonist, reduced POST after orotracheal intubation and compared with placebo.

## Methods

Written informed consent was obtained from 40 healthy young patients undergoing abdominal and pelvic surgery under general anaesthesia. There were no restrictions on recruiting the patients by type of surgery. The study was conducted in a prospective, randomized, placebo-controlled, and single – blinded manner. Patients with anticipated airway difficulty, history of preoperative sore throat and asthma, known sensitivity to study drug or recent anti-inflammatory medication were excluded from the study. Furthermore, patients with upper respiratory tract disease were also excluded from this study. Patients requiring more than one attempt for passage of tracheal tube were excluded from the study. Patients in whom extubation provoked bucking or coughing were also excluded from the study.

Presuming the incidence of POST to be 60%, the power analysis^17^ (taking α = 0.05 and β = 0.90) calculated a sample size of 20 patients in each of the two groups to show a 50% reduction in the incidence. Hence, we chose to enroll 20 patients in each group.

Premedication consisted of tablet alprazolam 0.25 mg orally three hours before surgery. Patients were randomly assigned (by means of a random number table) in a single-blind manner into one of two groups according to the agent used for gargle. Group C received drinking water 30 ml and Group K received preservative free ketamine 1ml (50 mg) in 29 ml of drinking water by the operation theatre nurse and asked patients to gargle with the preparation for 40 seconds after their arrival in the operation room. Anaesthesia was induced 5 minutes later. The patients could not be blinded because of the different tastes of the two preparations.

Standard non-invasive monitoring was done throughout the anaesthesia. Following preoxygenation, induction of anaesthesia was done with fentanyl 2mcg.kg^−1^ and 2 mg.kg^−1^ of propofol sufficient to obtund the eye-lash reflex, followed by atracurium 0.5 mg.kg^−1^ to facilitate orotracheal intubation with sterile polyvinyl chloride endotracheal tube (having low pressure cuffs) with an internal diameter of 7.5 mm for women and 8.5 mm for men. Tracheal intubation was performed by an experienced anaesthesiologist having experience of >3 years. The endotracheal tubes were lubricated with sterile water at room temperature. Immediately after intubation, cuff of the endotracheal tubes were filled with a volume of room air required to prevent an audible airleak. Anaesthesia was maintained with oxygen 33% in nitrous oxide, supplemented with halothane. Supplemental analgesia during surgery was provided with small doses of intravenous fentanyl. Residual neuromuscular relaxation with atracurium was antagonized with neostigmine and glycopyrrolate on completion of surgery. Oropharyngeal suction before extubation was done under direct vision to avoid trauma to the tissues, confirming that secretion clearance was complete^18^.

The patients were interviewed in a standard fashion by a blinded investigator at 4, 8, and 24 hours after the procedure. POST was graded on a four point scale (0-3): 0, no sore throat; 1, mild sore throat (complains of sore throat only on asking); 2, moderate sore throat (complains of sore throat on his/her own); 3, severe sore throat (change of voice or hoarseness, associated with throat pain^19^). Other side-effects, if any, were also noted.

To compare patient characteristics, including age, height, body weight, and duration of anaesthesia and surgery student's t-test was performed. The Mann – whitney U-test was used for multiple paired comparisons of counts in patients with POST. *P*<0.05 was considered statistically significant.

## Results

The study population consisted of 40 patients; 20 patients gargled with ketamine (Ketamine group) and remaining 20 patients gargled with only water (Control group). There were no significant differences in the groups in terms of age, height, body weight, gender distribution, or duration of anaesthesia (Table 1). There were no severe complications in either group.

**Table 1:** Patient Characteristics in the Control and Ketamine Groups (Mean ± SD)

	Control (n = 20)	Ketamine (n = 20)
Male: Female(n)	8:12	11:9
Age (yr)	36.7±12.3	37.5±12.5
Weight (kg)	55.1 ±6.0	56.4 ±8.0
ASA physical status(I/II)	16/4	17/3
Duration of surgery(min)	86.6±14.7	85.1±19.0
Duration of anaesthesia(min)	109.3±20.6	106.5±25.8

In Control group 17(85%) patients complained of POST at 4 hours, out of them 15(75%) patients had POST at 8 hours, which remained for 24 hours in 12(60%) patients (Table 2). However in Ketamine group, 8(40%) patients complained of POST at 4 hours. Out of them 7(35%) patients complained of POST at 8 hours and, which remained in 5(25%) patients for 24 hours, *P*<0.05 (Table 2). No significant differences in mild and moderate sore throat at 4, 8, and 24 hours were noted among the groups. The severity of POST was significantly lower in Ketamine group than in Control group, *P*<0.05. Overall, the number of patients in Control group had significantly more incidence of POST at 4, 8 and 24hours (85%, 75%, and 60%) than in patients having ketamine gargle(40%, 35% and 25%), *P*<0.05 (Table 2). No local or systemic side effects were observed.

**Table 2:** Comparison of severity of POST at 4,8 and 24h.(Data are presented as number(%) of patients.

	4 hr		8hr		24hr	
Groups	C(n=20)	K(n=20)	C(n=20)	K(n=20)	C(n=20)	K(n=20)
Grading of discomfort						
Mild	10(50%)**	7(35%)	8(40%)**	6(30%)	5(25%)**	4(20%)
Moderate	4(20%)**	1(5%)	2(10%)**	1(5%)	1(5%)**	1(5%)
Severe	3(15%)	0	5(25%)*	0	6(30%)*	0
Total no. of patients having POST	17*(85%)	8(40%)	15*(75%)	7(35%)	12*(60%)	5(25%)

* *P* < 0.05 between group comparison.

** *P*> 0.05 No significant differences in mild and moderate sore throats at 4,8, and 24 hours.

## Discussion

In the Control group, the incidence of POST 4, 8, and 24 hours after surgery was 85%, 75%, and 60% respectively (Table2). The reported incidence of POST is between 45 and more than 90%^4^,^5^,^18^,^19^. Our result in the Control group was consistent with previous findings. In our study, the incidence was significantly lower in the Ketamine group than in the Control group. There were no adverse reactions in the ketamine group. This is the first report of the efficacy of gargling with Ketamine in reducing POST in our country.

Several contributing factors for sore throat after surgery have been reported, including patient age, sex, large tracheal tube, cuffdesign, and intracuff pressure^20^–^24^. In our study, no correlation was observed between age, gender, duration of surgery and intubation.

Sore throat related to orotracheal tube might be consequence of localized trauma, leading to aseptic inflammation of pharyngeal mucosa. It may also be associated with oedema, congestion, and pain^2^,^25^. Reduction of this inflammation by ketamine gargling may be the reason for decreased in POST in our study. However, a peripheral and central action following systemic absorption cannot be excluded.

The antiinflammatory properties of ketamine have been shown against lung injury^26^. Moreover, ketamine has been shown to diminish the expression of inducible nitric oxide synthase^27^. Further, in an animal study, it has been shown that nebulized ketamine attenuated many of the central component of inflammatory changes^16^.

In this study, we identified POST as clinical anaesthesia outcome associated with routine surgery that is common and important to avoid, at least from the physician's perspective. Furthermore, we demonstrated that gargling with ketamine effectively attenuated POST, with no adverse reactions. Ketamine gargling might also reduce the incidence of complications associated with endotracheal intubation.
